# Interdisciplinary Collaborative Care to Manage Total Pain in Children with Cancer

**DOI:** 10.3390/children9040562

**Published:** 2022-04-14

**Authors:** Karen M. Moody

**Affiliations:** Section of Pediatric Palliative and Supportive Care, Department of Pediatrics, University of Texas MD Anderson Cancer Center, Houston, TX 77030, USA; kmoody@mdanderson.org

The pain and suffering of children with cancer became national news in the winter of 2000 with the publication of Wolfe’s landmark paper in the New England Journal of Medicine, “Symptoms and Suffering Children with Cancer” [1]. The paper drew attention to a suffering that our profession may have been avoiding, and it galvanized the birth of pediatric palliative and hospice care (PPC). PPC has since developed into a field positioned to address the pain and suffering of children with life limiting and life threatening disease. Subsequently, there has been an explosion of literature addressing pediatric palliative care clinical gaps, research needs, educational opportunities, program development guides, standards of care, and pain management strategies. The field has successfully moved the needle for children with cancer as depicted in a recent systematic review illustrating many positive impacts of pediatric palliative care [2]. Pediatric palliative care has improved pain and symptom management, quality of life, physical and emotional functioning, patient–provider communication, resource utilization, documentation of advance directives, and family satisfaction with care—all without any adverse effects on duration on survival [2]. As a matter of fact, in adults with lung cancer, early palliative care has been shown to lengthen survival [3]. Efforts are currently underway to establish clinical practice guidelines for pain and suffering in children with cancer by the International Pediatric Oncology Guidelines in Supportive Care Network [4]. Our group and others have published helpful approaches for treating terminal pain [5], chronic pain [6], and neuropathic pain [7] in children with cancer to provide clinicians immediate access to resources to address physical pain in their patients.

In this Special Issue “Total Pain Management in Children with Cancer,” the discussion of pain in Children with Cancer is expanded to reflect Dame Cicely Saunders’ definition of total pain with its inherent physical, social, spiritual, and psychological/emotional dimensions and includes the suffering of family members as well as patients [8]. The manuscripts herein represent the work from authors from palliative care, pediatric oncology, behavioral science, pediatric critical care, nutrition, psychology, pediatric and orthopedic surgery, nursing, child life, education, physical/occupational therapy, biostatistics, anesthesia, visual arts and music therapy. Harden et al. present common pain syndromes afflicting children with cancer during hematopoietic stem cell transplantation and concomitant management strategies [9]. Le et al. present a much-needed, evidence-based review of interventional pain management strategies in children with cancer and clarify when children may benefit from consultation with an anesthesiologist [10]. Revuri et al. provide a description of the pain experience of a series of children undergoing hemipelvectomy, generally indicated to cure cancer, but also a palliative intervention for pain [11]. They show that the pain and functional outcomes are excellent when pain is managed using a multidisciplinary, multimodal approach. Dr. Swartz and her study team describe the first reported enhanced recovery after surgery protocol for children with cancer with an aim to reduce opioid exposure, length of stay and surgical complications [12]. We look forward to future reports on the outcomes of this protocol.

The spectrum of symptom burden in adolescents with cancer undergoing stem cell transplant is measured by a novel assessment tool (the MD Anderson Symptom Index-adol) by Dr. Robert’s team and gives the readers an in-depth understanding of what these adolescents are experiencing both physically and psychologically [13]. Similar to adults with cancer, fatigue emerged as the most universal symptom, and one that is in need of better evidence-based treatment strategies. In an article by Sheikh et al., the authors provide a comprehensive review of sleep disturbances in children across the cancer trajectory, highlighting the adverse effects of impaired sleep on health-related quality of life, etiologies and risk factors for poor sleep and areas in great need of more research [14]. Dr. Tewari’s team presents a successful quality improvement initiative to improve mobility in children, adolescents and young adults undergoing hematopoietic stem cell transplant [15]. Given all we know about the benefits of exercise and movement in the general population, this initiative could lead to very exciting health benefits for this population.

Itzep and Roth report on the psychosocial challenges facing adolescents and young adults with cancer due to the interference the cancer experience has on normal developmental milestones in this age group, which can lead to distress and also post traumatic growth [16]. Stavinoha et al. describe direct and indirect barriers to successful educational attainment for pediatric brain tumor survivors and how clinicians can work with this population to overcome these barriers [17].

Finally, Cahalan and colleagues report how an interdisciplinary team can use collaborative legacy-building activities as a medium for emotional healing prior to end of life for children facing terminal cancer [18]. This is followed by Robert et al., who engaged bereaved parents to use narrative medicine as a teaching tool to deepen our understanding of child and parental needs when children are facing terminal illnesses [19].

The reality is that as long as cancer exists in pediatrics, pain cannot be eliminated. However, the suffering that results from pain and cancer can be alleviated to a great extent when clinicians use the four domains of physical, psychological, social and spiritual distress as a guide to the holistic assessment and management of total pain. As we enter the third decade of pediatric palliative care, it is abundantly clear that interdisciplinary collaborative care is the way forward to alleviating pain and suffering in children with cancer.
